# Prenatal Characteristics of Infants with a Neuronal Migration Disorder: A National-Based Study

**DOI:** 10.1155/2012/541892

**Published:** 2012-04-03

**Authors:** Estelle Naumburg, Bo Strömberg, Helle Kieler

**Affiliations:** ^1^Department of Women's and Children's Health, Uppsala University, Akademiska Sjukhuset, 751 85 Uppsala, Sweden; ^2^Department of Paediatrics, Östersund County Hospital, 831 83 Östersund, Sweden; ^3^Uppsala University Children's Hospital, 751 85 Uppsala, Sweden; ^4^Department of Medicine, Centre for Pharmacoepidemiology, Karolinska Institutet, 171 76 Stockholm, Sweden

## Abstract

The development of the central nervous system is complex and includes dorsal and ventral induction, neuronal proliferation, and neuronal migration, organization, and myelination. Migration occurs in humans in early fetal life. Pathogenesis of malformations of the central nervous system includes both genetic and environmental factors. Few epidemiological studies have addressed the impact of prenatal exposures. All infants born alive and included in the Swedish Medical Birth Register 1980–1999 were included in the study. By linkage to the Patient Register, 820 children with a diagnosis related to a neuronal migration abnormality were identified. Through copies of referrals for computer tomography or magnetic resonance imaging of the brain, the diagnosis was confirmed in 17 children. Median age of the mothers was 29 years. At the start of pregnancy, four out of 17 women smoked. Almost half of the women had a body mass index that is low or in the lower range of average. All infants were born at term with normal birth weights. Thirteen infants had one or more concomitant diseases or malformations. Two infants were born with rubella syndrome. The impact of low maternal body mass index and congenital infections on neuronal migration disorders in infants should be addressed in future studies.

## 1. Introduction

Development of the central nervous system (CNS) is a complex process, which requires integration of many cellular processes including neural stem cell proliferation, migration, and neuronal differentiation. The major neuronal migration occurs in humans between the 12th and the 24th weeks of gestation and results in the formation of the cortical plate [1]. However, the cortex continues to develop, and late migrations from the germinal matrix into the cerebral cortex continue until five months postnatally [1].

Classification of neuronal migration disorders is based on morphological criteria and includes schizenccphaly, porencephaly, lissencephaly, argyria, macrogyria, pachygyria, microgyria, and micropolygyria [2]. Pathogenesis of these malformations is multifactorial and includes genetic factors and environmental agents. In recent years the roles of several genetic mutations have been investigated. Few epidemiological studies have addressed the impact of prenatal exposures, although several maternal factors such as use of ethanol or drugs, viral infections, maternal diabetes, and untreated phenylketonuria can potentially influence the neuronal migration [3].

The aim of this population-based study was to describe perinatal characteristics in infants diagnosed with neuronal migration disorders aiming at identifying potential risk factors.

## 2. Material and Method

The infants included in the study were identified as those hospitalized with a diagnosis related to a neuronal migration abnormality and confirmed by a computer tomography (CT) or magnetic resonance imaging (MRI).

### 2.1. National Registration Number

All Swedish residents are assigned a unique 12-digit national registration number, which is used for official population-based registers. This number makes it possible to identify individuals and collect certain information within registers and also to link information between different registers [4].

### 2.2. The Patient Register

The inpatient register is held by the National Board of Health and Welfare, and it records information concerning hospitalizations, including date of admission and discharge and primary and secondary diagnoses together with the national registration number. From 1987 and onwards, the coverage is approximately 99% of all patients hospitalized in Sweden. All diagnoses are classified according to the current ICD classification [5].

### 2.3. The Swedish Medical Birth Register

The Swedish Medical Birth Register was established in 1973 and includes data on more than 99% of all births in Sweden. A standardized set of medical records is used by all antenatal care clinics and delivery units and at the examination of the newborn infant. Selected information from the records is computerized and forwarded to the register, which is held by the National Board of Health and Welfare. For all births, medical information on maternal demographic data, the reproductive history, maternal smoking habits, the pregnancy and delivery, and neonatal factors is included. The registration starts at the first visit to the antenatal clinic and is completed when the mother and newborn infant are discharged from the hospital [6].

The medical records are stored at local and regional hospital and department archives. The records, which include copies of referrals for radiologic examinations, were used to identify the children who had been examined with CT or MRI and to validate the diagnosis of a neuronal migration disorder.

### 2.4. Exposure Data

Information concerning factors that might influence neuronal migration disorders was obtained through the Swedish Medical Birth Register. For the mothers we obtained information about age, body mass index at first antenatal visit (BMI, calculated as maternal weight in kilograms at first visit to the antenatal clinic/maternal height times height), chronic diseases prior to index pregnancy, reproductive history (parity, years of infertility), and smoking in early pregnancy. BMI was classified according to WHO definition as underweight <18.5 kg/m^2^, normal weight ≥18.5 kg/m^2^ and <25 kg/m^2^, overweight ≥25 kg/m^2^ and <30 kg/m^2^, and obesity ≥30 kg/m^2^. Smoking was classified as no smoking, smoking 1–9 cigarettes/day, and >10 cigarettes per day. We also obtained information on maternal diseases during pregnancy and mode of delivery (noninstrumental and instrumental vaginal delivery and emergency and elective Cesarean section). As chronic diseases we included kidney failure, celiac disease, epilepsy, ulcerative colitis, diabetes mellitus, asthma, and systemic lupus erythematosus. The information was retrieved by the International Classification of Diseases as ICD-9 or ICD-10 codes, and all ICD-9 codes were manually transformed into ICD-10 codes.

For the infants we obtained information on sex, gestational age at birth, birth weight and birth length, and the Apgar scores at one and five minutes. Gestational age was primarily estimated from the start of the last menstrual period. Small for gestational age (SGA) was defined as birth weight more than 2 standard deviations (SDs) below the mean birth weight for gestational age and sex according to a Swedish birth weight curve [7]. Appropriate for gestational age (AGA) was defined as a birth weight between –2 SD and +2 SD and large for gestational age (LGA) as a birth weight more than 2 SD above the mean.

### 2.5. Selection of Cases

All infants born alive from 1980 through 1999 and diagnosed with a neuronal migration disorder according to MRI or CT scan sometime during this period were eligible for inclusion in the study. A total of 820 infants had been hospitalized with a diagnosis such as congenital myopathies and malformations of the CNS that could be related to a neuronal migration disorder (ICD 9: 359.1-359.24, 742.1-24, 759.23, and ICD 10: Q 040.0-89.9, G 71.2). We requested information on performed CT or MRI scans by a letter sent to all paediatric and radiological clinics in the country, and about half of the 47 clinics responded to our request. In 120 of the patients, a CT or MRI scan of the brain had been performed. The diagnosis of neuronal migration was confirmed by the local radiologist in 17 subjects, 9 boys and 8 girls, all of whom were included in the study.

As the study was purely descriptive, no formal testing was possible. For certain characteristics (maternal age, BMI, gestational age at birth, and birth weight), mean and/or median values are presented together with ranges.

## 3. Results

### 3.1. Maternal Characteristics

The mother's median age was 29 years, and most women were born in Sweden (Table 1). Four out of 17 women smoked at the start of pregnancy. One of the mothers was underweight with a BMI below 19 at the start of pregnancy, and none was obese. The mean BMI of the mothers at first antenatal visit was 21.3 (range 17.6–28.5). None of the mothers had a chronic disease prior to pregnancy. A malformation of the uterus was reported in one woman. There was one pregnancy complicated with an oligohydramnios and another with a polyhydramnios. Placental transfusion syndrome was reported in one pregnancy with a papyraceous fetus. Most deliveries were normal and noninstrumental (Table 1).

Other diagnoses, without any relation to the pregnancy, were also reported. One woman suffered from chronic multifocal osteomyelitis and necrosis and another was involved in a motorbike accident. Maternal care for (suspected) damage to the fetus by radiation was reported in one case.

### 3.2. Subject Characteristics

Nine male infants and eight female infants were born after mean 39.3 gestational weeks and with a mean birth weight of 3327 grams. All children were born as singletons and were AGA, and all had a full Apgar score.

Most infants were classified to have an undefined migrational disorder, a disturbed migration of the immature brain or a migrational disorder of unknown type. Thirteen infants had one or more concomitant disorders at birth. Congenital malformations of the spleen or the heart, Potter's syndrome, and facial asymmetry were reported, including malformation of the eye and lens, microphthalmus, and cleft palate. Hereditary haemolytic anaemia was reported in one infant. Two infants had convulsions and peripheral neuropathy.

Congenital infections were reported in three infants. Two infants had rubella syndrome, and one had congenital pneumonia. Two infants had skin problems at birth, and one infant suffered from obstruction of the intestine by gallstones or meconium (Table 2).

## 4. Discussion

All infants were born at term and of ordinary birth weight to healthy mothers with normal or subnormal BMI and after normal pregnancies. Most of the infants had a concomitant malformation, and two infants were diagnosed with congenital rubella infections.

A failure of normal migration may result in neurons accumulated in unusual areas [1]. Previously most of the neuronal migration abnormalities were diagnosed by autopsy. Nowadays the use of modern techniques of brain imaging, especially MRI, has dramatically improved the ability to detect these neurological disorders while the child is still alive. Over 25 syndromes related to disturbances of neuronal migration have been described, and they cover a large variety of pathologies and severity. The incidence of disturbances of the neuronal migration is not known, but is increasing in the pediatric population probably due to the use of MRI techniques for brain imaging [8]. 

## 5. Environmental Risk Factors

### 5.1. Nutritional and Other Factors Related to Maternal Health

Epidemiological studies and experimental data have demonstrated the importance of nutritional factors in fetal brain development and the deleterious effects of nutritional deficits [9–11]. BMI was recorded for 12 of the women in the study, and more than half of them had subnormal or near subnormal BMI at start of pregnancy. Since 1992 maternal BMI has been recorded in Sweden at the first antenatal visit, and an increase from 23 to 24 in mean BMI was noted between 1992 and 2002 [12]. One can speculate whether the generally lower BMI among these 12 mothers of infants with neuronal migration disorder, as compared to the general pregnant population might have influenced the fetal brain development. However, it was not possible to calculate the adjusted association between maternal BMI and risk of neuronal migrations disorder in this small descriptive study. The impact of maternal BMI at start of pregnancy and/or weight gain during pregnancy on the fetal brain development and neuronal migration needs to be evaluated in a larger study.

Different maternal conditions can interfere with fetal growth. It is well known that maternal diseases during pregnancy, such as diabetes and nontreated phenylketonuria, and conditions where the placental blood flow is reduced, such as preeclampsia, could interfere with the fetal brain development [1, 9, 13, 14]. Even a less severe maternal hypothyroidism during the first half of gestation might affect the fetal neurodevelopment and neuronal migration [15]. Few studies have addressed the impact on maternal health on specific migration disorders. In our study all of the mothers were healthy prior to the pregnancy. However, conditions related to an abnormal pregnancy and conditions which all can cause suboptimal growth conditions for the fetus were reported in four women.

Neuronal migration disorders have been described in humans and/or in animal models following in utero exposures to infections. Some viruses, such as herpes simples, cytomegalovirus (CMV), or HIV, are potentially capable to persist in a latent form within the central nervous system. Several studies have described the teratogenic effect of CMV infection and the relation to neuronal migration disorders [16–18]. Adverse impacts of maternal infections due to rubella, group B Streptococcus, cytomegalovirus, toxoplasmosis, and chorioamnionitis in the fetal brain are known. Fortunately successful rubella immunization has reduced risks of fetal complications [19].

Information on infections during pregnancy was missing in this study. However, two children were reported with rubella syndrome at birth, which is more than expected. The percentage of susceptible pregnant women with rubella was gradually reduced from 12% in 1975 to just below 2% in 1994. Rubella syndrome is very uncommon, and between 1975 and 1985, only a mean of two cases per year were recorded in Sweden. Since 1985, no child with the rubella syndrome has been registered [20]. It can be speculated that rubella had an impact on the neuronal migration in two cases in this study.

### 5.2. Toxic Agents

It is well known that maternal smoking increases the risk of intrauterine growth restriction and affects the fetal brain by inducing intrauterine hypoxia or by acting directly on the developing brain [21–23]. Smoking habits among women have changed over time in Sweden. During the 1960s as many as 25% of the women smoked, this was reduced to 14% in 2004 [24]. In this study 23% of the women smoked, which was more than in the general pregnant population during this time period.

## 6. Limitation of the Study

The detection of cases was difficult mainly because of our strict criteria for eligibility, which included a CT or MRI scan confirming the diagnosis. In 120 of all hospitalized children, a CT or MRI scan of the brain was preformed, and only 17 of them were confirmed with a neuronal migration disorder.

Despite several reminders, only 50% of all hospitals in Sweden responded to our request to send in CT and MRI scans. None of the hospitals actively rejected participation in the study. The cause of this dropout is not known but was evenly distributed between level I, II, and III hospitals over the country. It can be speculated that journals from the study period were not easily found, and the time for searching these journals was regarded as too expensive for the hospitals.

The study refers to a time of great medical advances, and the use of CT and MRI scan is more widely used today. The cases were identified through the national patient register, and the responding hospitals were evenly spread over the country. Considering the increasing use of CT and MRI scans, a study performed in more recent years might detect more children with neuronal migration disorder.

## 7. Conclusion

This descriptive study indicates that there might be an impact of low or subnormal maternal BMI before and during pregnancy, maternal infection, such as rubella, and maternal smoking on fetal brain development, including neuronal migration. The roles of maternal BMI and congenital infections should be tested in future analytical studies.

## Figures and Tables

**Table 1:** Maternal characteristics: maternal country of birth: 1: Sweden, 2: Mediterranean countries. Year of birth of infant with neuronal migration disorder. BMI: weight in kg/length in cmX2, “—”: missing information in length or weight. Smoking: 1: no, 2: 1–9 cigarettes/day, 3: ≥10/day, “—”: missing information. Delivery: Vag: normal vaginal delivery, Cs: Cesarean section, Ve: vacuum extraction.

Age	Origin	Year of birth of infant	BMI	Smoking	Delivery
29	1	1982	—	—	Cs
34	1	1982	19.6	1	Vag
28	1	1982	20.8	2	Cs
29	1	1986	21.7	1	Vag
26	1	1986	—	2	Vag
20	1	1987	17.6	1	Vag
23	1	1988	19.5	2	Vag
32	1	1988	—	1	Vag
30	1	1991	—	1	Vag
19	1	1991	19.7	3	Ve
25	1	1993	23.7	1	Ve
34	1	1993	19.8	1	Cs
37	1	1993	28.5	1	Vag
30	1	1994	20.2	1	Cs
31	2	1998	20.0	1	Vag
23	2	1998	24.4	1	Vag
35	2	1999	—	1	Vag

**Table 2:** Infant conditions: year of birth, type of migration disorder, and concomitant disease.

Year of birth	Type of neuronal migrations disorder	Other conditions	
1982	Micropolygyria	Undefined skin disorder	
1982	Unknown type	Obstruction of the intestine	
1982	Unknown type	Neuropathy	Polycythemia
1986	Unknown type	None	
1986	Micropolygyria	Undefined skin disorder	
1987	Unknown type	Malformation of the heart	
1988	Unknown type	Rubella syndrome	Convulsions
1988	Unknown type	Rubella syndrome	
1991	Unknown type	Hereditary haemolytic anaemia	
1991	Unknown type	Malformation of the spleen	Malformation of the heart
1993	Micropolygyria	Congenital pneumonia	
1993	Schizencephaly	Potter's syndrome	Facial asymmetry
1993	Lissencephaly	Mononeuropathy	Convulsions
1994	Unknown type	Microphthalmus, cleft palate	Malformations of the eye
1998	Unknown type	None	
1998	Schizencephaly	None	
1999	Unknown type	None	
